# Classification of domestic violence Persian textual content in social media based on topic modeling and ensemble learning

**DOI:** 10.1016/j.heliyon.2024.e39953

**Published:** 2024-10-29

**Authors:** Meysam Salehi, Shahrbanoo Ghahari

**Affiliations:** Department of Mental Health, School of Behavioral Sciences and Mental Health (Tehran institute of psychiatry), Iran University of Medical Sciences, Tehran, Iran

**Keywords:** Domestic violence, Social media, Ensemble learning, Topic modeling, Latent dirichlet allocation (LDA)

## Abstract

**Objective:**

Due to the importance of monitoring social networks to categorize domestic violence content and extract practical knowledge for conducting preventive interventions, as well as analyzing the extensive Persian textual content related to domestic violence generated in social networks following the COVID-19 pandemic, primarily, this research aims to create the best domestic violence Persian textual content classification model using topic modeling content at first and then combining algorithms using ensemble learning to achieve the best model performance.

**Method:**

By collecting Persian textual data using hashtags related to domestic violence equally and randomly from Telegram, Twitter, and Instagram networks between April 2020 and April 2023, the content were considered for topic modeling using the LDA algorithm. By extracting the probabilities of each topic for each document in our dataset, we considered the topic that had the highest probability to be a label for that document. Following feature extraction from labeled datasets, the Stacking and Voting ensemble learning methods were applied.

**Result:**

The analysis of 337,287 textual data revealed five topics: family crime news, war violence, women's rights, and violent reactions. Also, compared to the voting method, the stacking method performed better with 96.4577 precision, 96.4499 accuracy, 96.4499 recall, and 96.4475 F-score.

**Conclusion:**

According to the study findings, practical knowledge of the extracted topics can assist mental health centers in making preventive decisions. Moreover, the built model has the most efficient performance among the built models for the multi-class classification of DV texts in the Persian language for social media monitoring.

## 1 Introduction

In spite of valuable preventive and interventional activities and strategies over the years, mental health organizations still face numerous problems as a result of Domestic Violence (DV) in terms of industrial, societal, physical, psychological and health [1]. DV still affects approximately 27 % of 15–49 year olds around the world, regardless of age, gender, socioeconomic status, or educational level. Although DV can affect a wide range of people, women are the most vulnerable. One-third of women worldwide suffer from DV of some kind, making them the most affected gender [2].

It becomes even more relevant for countries in the East. South East Asia (38 %), and Africa (37 %) had higher regional prevalence rates of violence against women than other regions such as America (30 %) and Europe (25 %) [3]. In a meta-analysis conducted by Hajnasiri et al. (2016), among Middle Eastern countries, the prevalence of DV against women in Iran was estimated at 66 % [4]. Morever, according to the Iranian Statistics Center, 80,187 physical examinations were conducted by forensic doctors for spouse abuse claimants in 2019, with the majority of physical examinations belonging to women [5]. In addition, the increasing number of DVs against women in Iran as a result of the COVID-19 pandemic and home quarantines increases medical and mental health costs, leading to harmful consequences that Iranian society is still struggling with [6]. On the other hand, as a result of legal weaknesses and ineffective criminal regulations, many women choose not to speak out about the violence they experience. The truth about the relationship is revealed only after they have left the house since they are reluctant to share these experiences and violent behaviors [7]. This situation needs implementing effective strategies and decisions, especially immediate preventional actions at any level, impeding the rise of this type of violence and its harmful impacts on society.

In addition to traditional prevention and interventions including questionnaires, running awareness campaigns, building numerous safety houses throughout Iran, and providing comprehensive mental health services, Iranian researchers have identified social media as a wealthy research area where Iranian DV victims express themselves without fear of being judged and stigmatized [8,9]. In addition to providing people with an opportunity to seek assistance, these networks have also opened up new avenues for mental health services to be provided [10]. In social networks, users may post, share, and analyze a large amount of data (textual, visual, and audio), providing the opportunity for analyzing and extracting practical knowledge [11]. According to Chegeni et al. (2022), 88.5 % of Iranians use social media, with WhatsApp (68.4 %), Telegram (65.7 %), and Instagram (61.1 %) being the most commonly used [12]. The number of Iranian Twitter users increased from 4.65 % in 2017 to 8.69 % in 2018, nearly doubling. Furthermore, this network has been extensively studied by scientists around the world [13].

As researchers have encountered big data generated in social networks, they have turned their attention to using novel analytical techniques and methods like artificial intelligence and Machine Learning (ML) methods to classify and predict DV-related content, aiming to extract practical information.

ML is an artificial intelligence technique that uses data and hidden patterns to predict future outcomes. A variety of ML approaches can be used to solve a particular problem, including supervised, unsupervised, and semi-supervised learning, as well as deep learning and Ensemble Learning [14]. ML techniques to analyze DV content in social networks have shown promising results in recent studies [15,16]. Social media platforms like Twitter and Reddit, as well as other sources such as professional notes and national databases, have been analyzed using data mining techniques and artificial intelligence algorithms [17,18]. They have used supervised and unsupervised ML methods, such as Random Forests (RF), Support Vector Machines (SVM), and Latent Dirichlet Allocation (LDA), to detect and classify DV-related posts, as well as topic modeling [16]. Despite the widespread use of ML algorithms in the field of mental health [19], it has not been long since the first research used ML algorithms in the field of DV classification and diagnosis globally.

In the first study in the field, 8856 posts and 28,873 comments, collected from 2014 to 2017, were analyzed by Subramani et al. (2017) using ML algorithms. Modeling was done by manually preparing 625 posts with suggestions and 510 posts with abuse tags and a Linguistic Inquiry and Word Count (LWIC) package was used to preprocess the English language. Then Term Frequency-Inverse Document Frequency (TF-IDF) was used to extract the features. In another study by Subramani et al. (2018), Deep learning was used for the first time in the field to identify critical DV posts on Facebook. A total of 750 posts were classified as critical, and 1310 posts as uncritical. After extracting text features using the Word2Vec model, deep learning algorithms such as CNNs and RNNs were used to model the text. Up to 94 % of labels can be predicted using deep learning algorithms [18]. Since then, researchers have focused their attention on the application of data mining methods such as ML to data related to DV.

In a recent study by Chen et al., in 2023, because traditional social survey methods underestimate the incidence rate of intimate partner violence due to underreporting by victims, ML algorithms were applied to predict the incidence rate of intimate partner violence in China using data from the Third Wave Survey on the Social Status of Women in China (TWSSSCW 2010). The random under-sampling ensemble method and the random forest algorithm were used to impute missing data. Analysis of the complete data showed higher incidence rates of physical violence (7.10 %), verbal violence (13.74 %), and cold violence (21.35 %) compared to the original dataset [20].

While ML methods are now commonly used to analyze DV content in global studies, especially English textual content, few studies have investigated Persian textual DV content on social media. Among Iranians, Persian is the first language they speak, and they use it extensively in social networks. The focus of text mining methods during studies related to DV has been clearly on the English language [21]. As a new language, Persian textual content analysis may provide new insights into this area. The first reason is that topic modeling results may be affected by different cultural contexts. Secondly, probabilistic computer models can be affected by language, which is a culturally loaded concept.

To the best of our knowledge, for the first time, Persian DV textual analysis based on DV-related hashtags drew the attention of Iranian researchers like Alami et al. (2020) and Ebadi-Jalal et al. (2021). These two studies were not based on ML, but they were the first to focus on the importance of social network data in examining the experiences of Iranian DV victims using hashtag analysis. In 1028 comments on related posts on Instagram, Ebadi-Jalal et al. categorized the structure of DV-related conversations [8]. Furthermore, Alami et al. examined the hashtag #Romina_Ashrafi and Instagram users' reactions to her murder. 33,740 posts were analyzed to identify the top posts as well as the most engaged and most visited content and users [9].

But in the field, recent research in 2023 was the first to predict Persian risky textual content using ML algorithms. Among 53,105 tweets and Instagram captions collected from Twitter and Instagram platforms, 1611 were selected at random and manually categorized into critical (229) and uncritical (1382). By implementing several ML algorithms, The Naïve Base model, with an accuracy of 86.77 % was the most accurate model among all ML models for predicting critical Persian content pertinent to DV on social media. In the context of Iranian DV studies on the Persian language, the study was the only one to develop a ML model to predict risky Persian DV content [22].

There are some significant gaps in the literature that led us to conduct this particular research. A primary gap was the lack of topic modeling on DV related content in Perisan. In fact, there was no descriptive analysis or research to provide practical knowledge regarding such a significant amount of content published on social media. Furthermore, no research has focused on developing a classifier model, while its cost-saving capabilities, automatic nature, and high precision detection of critical content in the field were undeniable in eliminating significant healthcare barriers that the Iranian healthcare system is experiencing.

The primary motivations for conducting this research were to eliminate the limitations of previous research, including the limited number of labeled datasets, the lack of combining several models using ensemble learning in order to achieve performance above 86 % accuracy [22], the lack of a study to determine topics that are most commonly discussed in DV Persian content on social networks, as well as the lack of a model for categorizing this content into multiple classes.

Thus, In the current study, our main purpose is to step aside further to achieve a more accurate model by modeling on more collected Persian textual data through LDA topic modeling to extract the most important topics discussed among Iranians on social media and then utilizing ensemble learning to develop the most effective Persian textual DV content classification model through the use of the method and a robust labeled dataset. As a result, several obstacles Iranian mental health organizations face when monitoring Persian DV content on social media were eliminated by accurate classification of this content. As a result of modeling the Persian language in the field and creating robust labeled datasets, it was possible to eliminate resource limitations for datamining Persian textual content. Therefore, this research has three main objectives.

1. Topic modeling of Persian content related to DV in social networks to reach the main topics of interest to people in this area and gain practical knowledge through the analysis of large amounts of data.
2. Labeling the existing dataset according to the maximum probability of the topic extracted from each document using the LDA model
3. Using ensemble learning to model existing labeled datasets in order to develop the most accurate model

## 2 Methods

We gathered all the data for this study from public tweets, Instagram posts, and Telegram public channels in order to maintain ethical considerations. In addition, the ethical committee at the Iranian University of Medical Sciences approved the study (ethical code number: IR.IUMS.REC.1401.008). Analyzing and modeling were conducted using Python version 3.12.2. A number of factors contributed to the selection of this program, including its ease of use, popularity, application to models using ML algorithms, the ability to call Persian language processing libraries, and the availability of various resources on the Internet for resolving coding errors. The main libraries used in this research in Python include Pandas, Metplatlib, Genism, Nampay, Hazm, and Parsivar.

### 2.1 Data gathering

Three social networks commonly used among Iranians, Instagram, Twitter, and Telegram, were chosen as the data collection medium for this study due to their textual nature, their popularity among Iranians [12], and their capability to utilize hashtags to collect data from these networks. We have chosen not to use other social networks, such as YouTube, and Facebook, and Iranian social networks such as Soroush and Rubika, since the nature of their data (video and audio) is not consistent with the objective of this research (work on the text). In addition to their limitations in the data collection process, there was a lack of hashtag usage similar to what was observed on Twitter, and it was also similar to the lack of analysis in previous studies in the field.

In this study, hashtags related to DV in Iran were used as the basis for data collection. All hashtags collected in previous research were considered and followed in this study [22]. Finally, the hashtag data from Twitter, Instagram, and Telegram was collected randomly over the last three years from April 2020 to April 2023. We chose this period of time because there were many DV movements in Iran, and their hashtags became trending on social media during this period. DV-related hashtags were also included, as were tweets, captions and Telegram channels' textual data about DV against women and its various forms. Meanwhile, tweets, captions and Telegram channels' textual data containing content related to violence against the elderly, violence against children, violence against men, and tweets, captions and Telegram channels’ textual data that appear to have been generated by bots are excluded. In the dataset, all data without a name, tweet, caption, biography, or photo was considered bots.

### 2.2 Preprocessing

In spite of the difficulties associated with preprocessing and cleaning Persian in natural language processing due to the presence of ironies, proverbs, and stuck forms of words and letters, Python is currently being used to process Persian language using the Parsivar and Hazm Persian libraries, which are specifically designed for Persian language processing [23,24]. Following the collection of the data necessary to extract the cleaned data, missing data were removed from the dataset. Due to the common alphabet between Arabic and Persian languages, the Arabic-language data were removed from the dataset, and only the Persian-language data was collected and considered. Then, characters such as letters, URLs, Persian StopWords, English letters, and Emojis were removed, as well as symbols such as (!_#@%"%/%[/]a-zA-Z//?: = ). Also, tokenizing, stemming, and lematizing operators were applied to the datasets in order to identify the roots of words and to remove duplicate and redundant forms from those roots. Furthermore, after all of the cleaning, redundant forms of verbs that remained in the dataset were removed using loops.

### 2.3 Topic modeling with LDA

Latent Dirichlet Allocation (LDA) is widely used in information retrieval and text mining. LDA uncovers latent semantic structures within a set of textual documents by using a probabilistic model [25]. It has been applied to a variety of fields, including web content analysis, news analysis, and language teaching research. Classification, clustering, and topic coherence measurement have been demonstrated to be effective with LDA [26]. Since LDA is an unsupervised algorithm, it is particularly suitable for unlabeled data, such as social media data collected on DV. As a result of LDA's flexibility, it is able to identify topic patterns even when the texts are sparse. On the other hand, It is common for textual data to be sparse, but preprocessing techniques such as removing stop words and stemming are helpful in reducing this problem. In addition, previous studies have demonstrated that LDA has consistently produced reliable results when applied to Persian social texts [27].

To label each document after extracting the main topics of the data, the LDA method is used in the current study due to the large volume of data and the extraction of their main topics. As a first step, the dictionary of main vectors was entered into the model using the *Dictionary* and *doc2bow* functions. Then, to identify the best number of topics, we considered a range of 2–10.

An important step in determining the LDA algorithm's results is determining the optimal number of topics (K-value). However, although several techniques have been implemented in Python libraries to estimate K-values [28], there is no standard method available for determining K-values. Choosing this range was based on the fact that we were looking for general topics in our corpus so that we could perform better with ML algorithms, and avoid having too many classes for labeling. We also considered the values of 0.01, 0.1, 0.3, 0.5, 0.7, and 0.9 for each of the LDA model hyperparameters like Alpha and Beta in order to achieve the most appropriate values. For each of the hyperparameters, we evaluated the model's performance with the coherence value.

In LDA, a coherence value represents the semantic coherence of a document or of a segment within a document. It is used to determine the degree of topic-relatedness and consistency in words associated with a particular topic. Higher coherence values indicate stronger semantic connections between words within a topic, whereas lower coherence values indicate weaker connections [29]. Upon reaching the best hyperparameter value for the number of topics, alpha and beta values, topic modeling was performed using LDA. The relevance of the extracted topics was checked by the authors and labels based on the words associated with each topic were assigned.

Then, using the model, we extracted the probabilities associated with each topic for each data, and the highest topic probability was added as a label to that data. All of the data was labeled and categorized in this manner in order to prepare datasets for training ML algorithms.

### 2.4 Feature extraction with LDA and TF-IDF an PCA

In order to extract features for modeling, in addition to the LDA method, which obtained topic probabilities for each document in the previous step, the TF-IDF method was also employed to extract features, as well as enrich the feature vector for modeling. The TF-IDF technique is used to weight terms in text analysis. It considers both the frequency of a term in the document (TF) and its rarity across all documents (IDF). There are many uses for it, including hate speech classification and hoax detection on Twitter [30,31]. Based on existing studies, we selected the method due to its significant performance in regards to Persian textual content [22].

In addition, we applied the Principal Component Analysis (PCA) method to reduce effective features extracted by TF-IDF to achieve lower dimension space and lower model complexity. In text classification, PCA is widely used for feature extraction and dimension reduction [32]. Based on variance maximization, it maps the original high-dimensional feature space into a lower-dimensional space. Through PCA, more representative features can be extracted and weaker ones filtered out to improve text classification accuracy and efficiency. Additionally, PCA based on similarity/correlation rather than covariance can improve text classification performance, particularly in high-dimensional feature spaces [33].

First, 5000 features with Ngrams (1,1) were extracted from the corpus using the TF-IDF method, and then PCA method were applied to TF-IDF features for dimension reduction from the Sklearn library. The final feature vector was created by combining the probability values of each topic within each document as well as the features extracted from the PCA technique, providing the feature vector required for ensemble learning modeling. Since the current dataset contains labels and features extracted from LDA and PCA, to better learn the algorithms, an equal number of data from each label was randomly selected to ensure that the classes would be balanced. Afterward, the feature vectors were normalized using MinMaxScaler to ensure that all values fall within a certain range.

### 2.5 Modeling with ensemble learning

In spite of the fact that we used different ML algorithms in the previous research [22], due to the large number of data and their dimensions in the current study, as well as in order to achieve the highest modeling performance, we decided to use ensemble learning. To improve classification accuracy and performance, ensemble learning combines multiple models. It reduces overfitting, enhances generalization, and handles high-dimensional data effectively [34]. Having divided the dataset into two parts, training (80 %) and testing (20 %), we considered ensemble learning algorithms Support Vector Machines (SVM), Random Forests (RF), and K-Nearest Neighbours (KNN) based on the classification problem that was presented in this study. Additionally, we evaluated the performance of two popular ensemble learning methods [34].

In ML, ensemble learning techniques such as Voting and Stacking are often used. Voting involves combining multiple models to make predictions, either by taking the majority vote or by assigning weights to each model's prediction [35]. On the other hand, stacking involves training a meta-model based on the outputs of multiple base models. Numerous studies have explored both voting and stacking. The use of these techniques has shown promising results in the improvement of prediction performance and the reduction of decision-making errors [36].

Thus, considering algorithms SVM, RF, and KNN as estimators in voting method and SVM, RF, and KNN as estimators with the Logistic regression (LR) algorithm as final estimators in stacking method, modeling was done with the two methods. Then, a k-fold cross-validation method was applied to all parts of the existing dataset to avoid overfitting. The data is divided into K subsets using this method. A subset of these is used for validation, followed by a subset of k-1 for training. K datasets are used for training and validation exactly once each, with every dataset being used K times. The average result of these K validations is used as a final estimate [37]. We applied stratified k-folds to ensemble learning methods with a 5-fold setting and then averaged the accuracy of all five times to identify the algorithm with the highest mean accuracy.

### 2.6 Models’ performance evaluations

As classifier evaluation metrics, Precision, Recall, F-score, and Accuracy are chosen. Previous studies have extensively examined these metrics to determine models' performance [17,38]. To evaluate memorizing the model rather than learning from the training data, the average test accuracy of the models was reported on test data.

## 3 Results

Following the application of entry and exit criteria and the removal of missing data from the dataset, 337,287 Persian textual data were extracted equally and randomly from Telegram, Instagram, and Twitter from April 27, 2020, to April 27, 2023. Following preprocessing, the number of words decreased from 16,697,613 to 7,381,512.

**Fig. 1:**
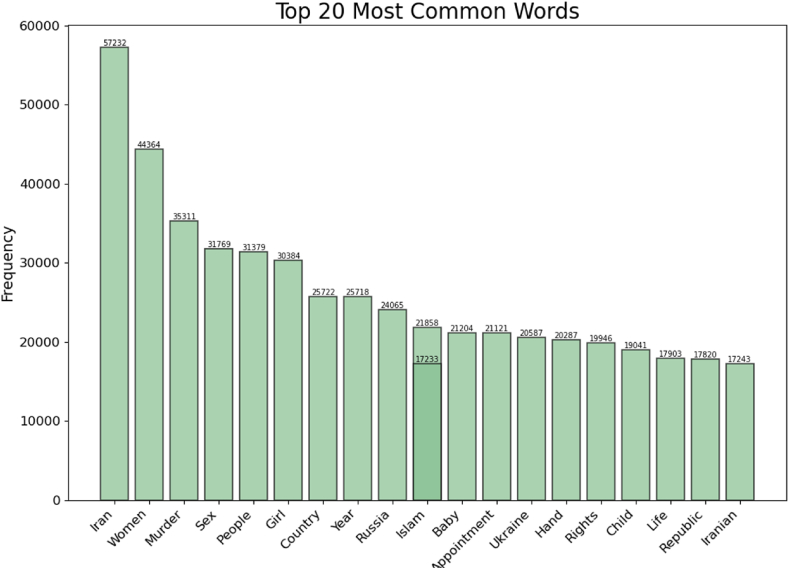
**Top 20 Most Frequent Words**. This bar chart shows the frequency of the 20 most common words in the dataset, with ‘’Iran’’ having the highest frequency followed by ‘’Women’’, “Murder”, and “Sex”.

Based on Fig. 1, the top 5 most frequent words were “Iran” with a frequency 57232, “Women” with frequency 44364, “Murder” with frequency 35311, “Sex” with frequency 31769, and “People” with frequency 31379.

According to the method section, different values of each hyperparameter were evaluated with coherence values in order to determine the best hyperparameters for the LDA model.

**Fig. 2:**
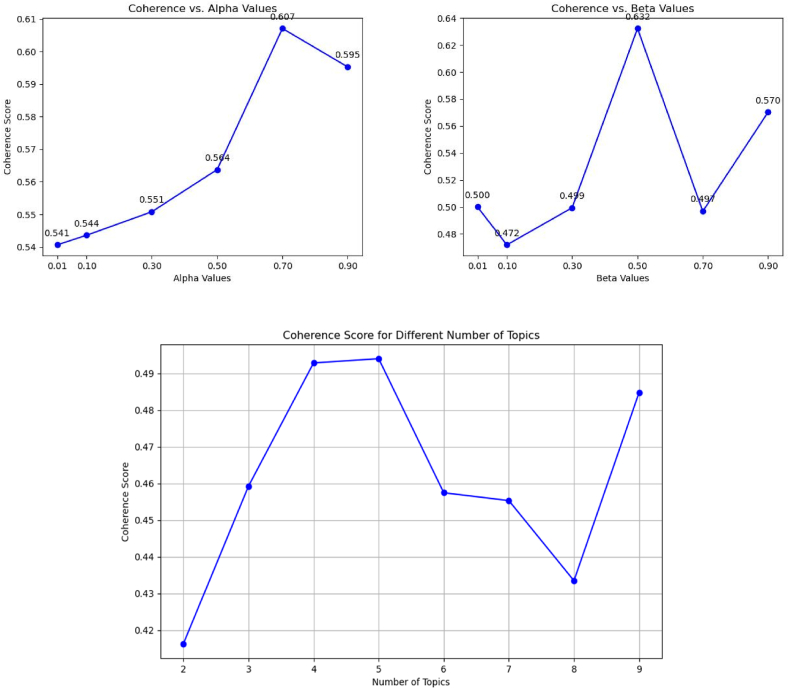
**Coherence Values for Different Hyperparameters**. Coherence scores are plotted based on alpha, beta, and number of topics, illustrating the impact of hyperparameters.

Based on Fig. 2, the best values for each parameter were 5 topics with a coherence of 0.5, 0.7 alpha value with a coherence of 0.6, and 0.5 alpha value with a coherence of 0.63. Based on these hyperparameter values, LDA modeling was conducted.

**Fig. 3:**
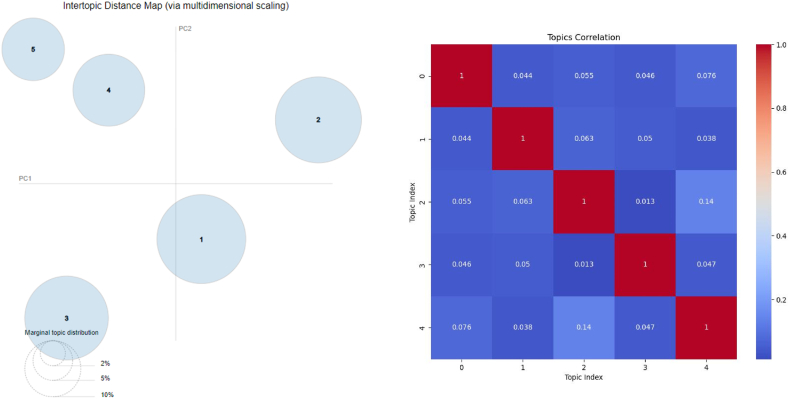
**The topic model performance.** The left graph displays the distribution of topics in 2D space, and the right heatmap illustrates the correlation between topics.

With the help of the pyLDAvis and topic coherence heatmap, Fig. 3 illustrates the intercoherency of topics and the separation of each topic from the other.

On the basis of the figure, topics 2 and 4, with a correlation of 0.14, followed by topics 0 and 4 with a correlation of 0.076, had the highest correlation. Also, the LDA model has performed well in separating topics from each other. According to the Figure, all topics are clearly separated. Based on the Intertopic Distance Map and Topics Correlation Heatmap shown in the figure, it appears that the topics do not overlap significantly. In the Intertopic Distance Map, topics are represented by circles that are spaced apart, indicating their separation. In addition, the Topics Correlation Heatmap shows low correlation values (e.g., 0.044, 0.063), which suggests minimal overlap. As a result, the generated topics are generally well-separated and there is no overlap between them.

This model had an overall coherence value of 0.52 and a Perplexity level of −9.41, which indicates that it performed well in extracting the key topics in the text. Table 1 shows the words related to each topic.

**Table 1:** Topics most important related words.

Topic	Label	Topic most important related words	Topic most important related words (translated to English)	Topic coherency	Topic percentage
1	Family crime news	قتل، دختر، ناموسی، کودک، سال، پدر، ناموس، کشی، اعدام، نفر، زندان، خانواده، مادر، خبر، شکنجه، خواهر	Murder, daughter, honorable, kid, year, father, honor, killing, execution, person, prison, family, mother, news, torture, sister	50.41	20.03
2	War Violence	روسیه, اوکراین, ایران, کشور, مردم, پوتین, آمریکا, حمله, خاک, نظامی، اسلامی، یمن، محکوم، حمایت، دفاع	Russia, Ukraine, Iran, country, people, Putin, America, attack, soil, military, Islamic, Yamen, convicted, support, defense	1.28	15.36
3	Women's Rights	زنان, جنسی, حقوق, قرار, خشونت, جامعه, دلیل, استفاده, صورت, قانون، حق، قانونی، حریم، رابطه	Women, sextual, rights, appointment, violence, society, reason, use, form, law, right, legal, privacy, relationship	27.44	23.32
4	Violent reactions	بدکاره, خدا, روسپی خانه, مادران، مردم، محارم، لواط، حرام	bitch, God, brothel, pussy, ass, mothers, people, Indecent, sodomy, forbidden	33.41	7.26 %
5	Social issues	بچه, حرف, آدم, دوست, دست, بد, متجاوز, درست, فرق, مشکل	Child, word, person, friend, hand, bad, aggressor, right, difference, problem	38.30	34.03

This table shows the key related words for each topic in Persian and English, along with topic lables, coherence scores, and dataset covereage percentages.

In order to assess the relevance and meaningfulness of the generated topics, we conducted a human evaluation after the topic modeling process. Topic coherence and relevance to the documents for each topic were considered by two annotators with expertise in DV (Appendix 1). Topic coherence of topics was rated by Annotator 1 with a mean score of 4.0 and a standard deviation of 1.0 by answering 5 Likert options, indicating moderate consistency. While Annotator 2 had a slightly higher mean score of 4.2 and a standard deviation of 0.84, their ratings were less variable. In terms of topic relevance, Annotator 1 assigned an average score of 4.4 with a standard deviation of 0.89, indicating relatively consistent judgments. Additionally, Annotator 2 provided a slightly higher mean score of 4.6 with the same standard deviation of 0.89 as Annotator 1. Human annotators rated topic coherence more positively than the internal model, with scores between 4 and 5, whereas the model's overall coherence value is 0.52, which indicates moderate topic coherence. Moreover, the model's perplexity score of −9.41 indicates that it handles complex data well.

Through the LDA model, the probabilities of topics for each document in the dataset were extracted, and the topic with the highest probability was labeled as a data tag (Appendix 2). As a result, all the data were labeled.

Based upon the labeled dataset, 50,000 data points were randomly selected from the dataset and an equal number of 10,000 data points were assigned to each of the five classes in order to ensure that the classes were balanced.

As a result of extracting features using the TF-IDF approach to a number of 5,000, the PCA method was used to determine the most important features by reducing the dimensions. The Cumulative Explained Variance Ratio was used to determine the optimal number of PCA components. As a result, we set the number of PCA components equal to the number of features obtained from the TF-IDF method (5000) to determine which PCA components contain more accumulated variance, as well as to identify features that are common or whose separation of classes does not change much.

**Fig. 4:**
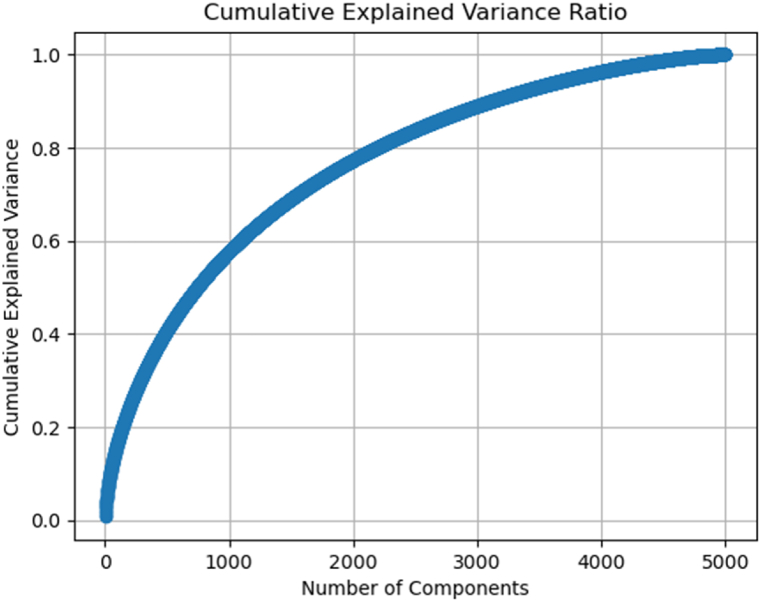
Different number of components up to 5000 vs Cumulative Explained Ratio.

Based on Fig. 4, it's evident that after approximately 3500 components, the curve flattens significantly. This means that adding more components beyond 3500 contributes very little to explaining additional variance. Hence, selecting 3500 components seems reasonable, as beyond this point, the explained variance increase is marginal. By choosing 3500, almost all the important variance was captured, while avoiding unnecessary complexity in the model by including too many components with minimal added value.

The 3500 components and 5 topic possibilities were used to construct a feature vector with dimensions of 3505∗50,000 for modeling purposes. After modeling with the feature vectors, a 5-fold cross-validation procedure (each fold = 10,000) was performed. The mean metrics are shown in Table 2.

**Table 2:** Models metrics after 5-fold cross validation.

Ensemble learning methods	Mean precision (%)	Mean recall (%)	Mean F-score(%)	Mean train accuracy(%)	Mean test accuracy(%)
Voting	96.2650	96.25	96.2495	99.85	96.25
Stacking	96.4577	96.4499	96.4475	99.98	**96.4499**

This table highlights the performance of two ensemble learning methods (Voting and Stacking) after 5-fold cross-validation, showing metrics such as precision, recall, F-score, and accuracy on both training and test sets.

On the basis of Table 2, the Stacking method is more effective than the Voting method. In order to avoid overfitting the models, we used class balancing and cross-validation due to the high dimensionality of the feature vector. Based on a comparison of train and test accuracy values (about 3 units), it can be concluded that the models are well-trained.

**Fig. 5:**
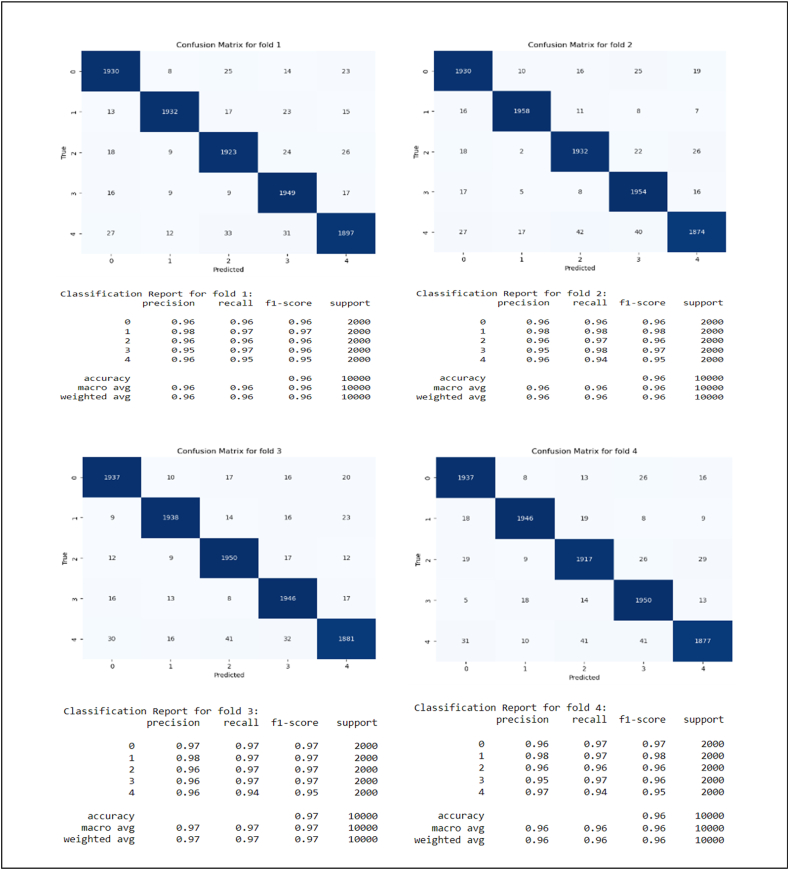

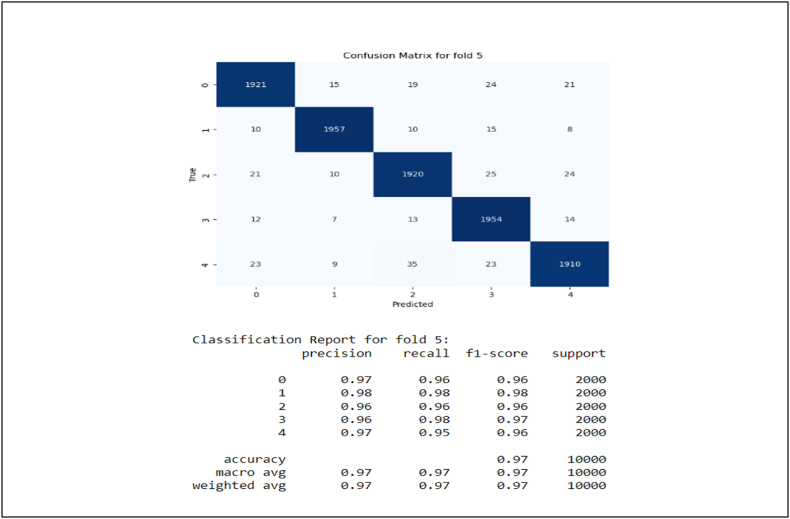
**Stacking Method Performance on Each Fold Test Data**. Confusion matrices for five different folds display the model's performance in terms of precision, recall, and F1 score across each test fold.

Fig. 5 also demonstrates confusion matrix and classification reports in each fold of cross validation.

According to the figure, In all folds, the model demonstrated significant accuracy between 0.96 and 0.97. Moreover, Precision, Recall, and F1-score were almost the same and higher than 0.96. For example, in fold 1, the accuracy for different categories changes from 0.95 to 0.98, and in fold 2, a similar accuracy can be observed. There were typically fewer than 30 incorrect predictions per category out of 2000 samples, indicating high model accuracy. Across all validation steps, the weighted total of all features in folds remained between 0.96 and 0.97, showing stable performance.

## 4 Discussion

At all stages of this study, we sought to develop a model that could accurately classify Persian textual content related to DV. First, we collected the Pesian textual data published on Telegram, Twitter, and Instagram during the three years of 2020–2023, and then we used LDA topic modeling to identify the most important topics discussed by Iranians in this field in social networks. Then, we assigned a label to each data based on the most likely topic through LDA topic probabilities. In the next step, we created a feature vector for ensemble learning modeling by using the LDA and TF-IDF feature extraction methods, as well as the PCA dimension reduction method. As a result of combining algorithms SVM, RF, KNN, and LR, as well as two the Voting and the Stacking methods, the best model was developed.

As a result of topic modeling on gathered data from the social media, All DV Persian textual content is summarized in Table 1 under the following five topics: Family crime news (20.03 %), War Violence (15.36 %), Women's Rights (23.32 %), Violent reactions (7.26 %), and Social issues (34.03 %). As shown in the table, social issues account for the majority of discussions, while violent reactions account for the smallest percentage. Despite the similarities between the topics of family crime news and social issues in this study and those of DV news and social movements and awareness in Xue et al. [39], there are also different topics which seem to arise from Iranian geographical and socio-economic conditions. One manifestation of the different conditions can be the fact that 15.36 % of the volume of conversations is devoted to topic 2 (war violence) and 7.26 % is devoted to topic 4 (violent reactions).

Moreover, as a result of the collection of data from Twitter, Instagram, and Telegram instead of just Twitter, as well as the lengthy extraction process, compared to studies that collected data exclusively from Twitter [40] and around for over a year [22], LDA topic modeling and text classification results are more generalizable.

According to Table 2, with respect to precision, recall, F-score, and both training and test accuracy, Stacking outperforms Voting. Stacking achieves 96.4499 % test accuracy while Voting achieves 96.25 %, showing better generalization. Moreover, there is no overfitting in their performances as the differences between train and test accuracy are not significant. Accordingly, the Stacking method with 96.4577 precision, 96.4499 accuracy, 96.4499 recall, and 96.4475 F-score performed the best for the classification of textual data related to DV in the Persian language.

Research at the international level has focused on English and then on Chinese content [21], and Iranian DV studies often examine the effectiveness of different treatments for DV victims [41], the identification of factors [42], and the prevalence of this type of violence [43]. In this situation, developing a DV Persian textual content multi-labeling model with such high performance will not only expand knowledge in the field of Persian language text mining but will also eliminate the current research gap on the national and international levels.

As compared to previous research on Persian content [22], the performance metrics in the model constructed in this study have increased by 10 units, so that accuracy has almost reached 96 % from 86 %. Although the model's performance is lower than the model developed by Liu et al. (ACC = 97 %) [44], it has a higher performance than the model developed by Garrett et al. on the English language (F = 0.78 %) [45], a higher performance than the model developed by Chu et al. on the Chinese language (ACC = 0.94 %) [46], and a higher performance than the model developed by Subramani et al. on the English language (ACC = 0.91 %) [47].

We faced a research gap when using topic modeling on DV Persian content, despite topic modeling on Persian data being a common method, particularly in COVID-19 content analysis [40]. Based on measuring different values (Fig. 2), the most suitable hyperparameters have been identified, resulting in high coherence, low complexity, internal correlation, and separation of topics (Fig. 3) in line with similar studies [40]. However, in the study by Xue et al., no information was provided regarding the best method of selecting topics [39].

### 4.1 Applications and limitation

In order to extract practical knowledge from a massive amount of unstructured data more efficiently, Persian text related to DV can be monitored in social networks more effectively than ever before using the developed LDA topic model and multi-class classification with the ensemble learning model to facilitate mental health service centers, preventive policies, and online interventions.

Fig. 5 shows a practical example of Persian textual data classification in DV. On the basis of 10,000 test data, the model performs at 96 % in each fold. From now on, the model can be used in mental health settings to monitor and categorize the contents of DV Persian textual data in Iran and around the world. Alternatively, the current labeled dataset may be used for future studies in order to enhance knowledge in this area and to conduct research.

The most important limitation of this study was that we attempted to reduce the volume and complexity of modeling by using random selection and creating a 50000 subsample from the main dataset due to time-consuming and expensive computational issues at different stages of this study, especially during modeling. Although this method has the advantages of reducing the time and complexity of the model, future studies, assuming they are not bound by administrative limitations in terms of hardware and software in modeling and are able to perform topic modeling or ML modeling on all of the data, may be able to achieve more significant generalizations.

## 5 Conclusion

This study aimed to develop a model for categorizing Persian textual content related to DV in social media, along with topic modeling of this content for practical purposes. This research revealed that this content can be divided into five topics, including Family crime news, War Violence, Women's Rights, Violent reactions, and Social issues. It is also possible to monitor all relevant content in social networks on a national and international level by the built model using stacking method in ensemble learning. This method has performed significantly better than the Voting method in the automatic classification of this content.

Through the use of this built model, all relevant content in social networks in the field of mental health can be monitored at the national and international levels. This eliminates a significant portion of the research and operational costs and gaps associated with monitoring DV content in Persian.

## Decleration section

The work described has not been published previously except in the form of a preprint, an abstract, a published lecture or academic thesis.

The article is not under consideration for publication elsewhere.

The article's publication is approved by all authors and tacitly or explicitly by the responsible authorities where the work was carried out.

If accepted, the article will not be published elsewhere in the same form, in English or in any other language, including electronically without the written consent of the copyright-holder.

## CRediT authorship contribution statement

**Meysam Salehi:** Writing – review & editing, Writing – original draft, Visualization, Validation, Supervision, Software, Resources, Project administration, Methodology, Investigation, Funding acquisition, Formal analysis, Data curation, Conceptualization. **Shahrbanoo Ghahari:** Writing – review & editing, Supervision.

## Data and code availability statement

Data will be made available on request.

## Declaration of competing interest

The authors declare that they have no known competing financial interests or personal relationships that could have appeared to influence the work reported in this paper.

## Footnotes

Appendix A Supplementary data to this article can be found online at https://doi.org/10.1016/j.heliyon.2024.e39953.
