# Supplementary material for: Classification of domestic violence Persian textual content in social media based on topic modeling and ensemble learning
Source: Heliyon. 2024 Oct 29;10(22):e39953. doi: 10.1016/j.heliyon.2024.e39953 (PMC11583712; doi:10.1016/j.heliyon.2024.e39953)
Supplement: Multimedia component 1 [file mmc1.docx]

# Appendix 1. External human evaluation result (5 Likert options)

| Topic | Topic coherence (Labeler 1) | Topic coherence (Labeler 2) | Topic-Document Relevance (Labeler 1) | Topic-Document Relevance (Labeler 2) |
| --- | --- | --- | --- | --- |
| Topic 1 | 4 | 4 | 5 | 5 |
| Topic 2 | 5 | 5 | 5 | 5 |
| Topic 3 | 5 | 4 | 5 | 5 |
| Topic 4 | 3 | 5 | 3 | 3 |
| Topic 5 | 3 | 3 | 5 | 4 |
