# Supplementary material for: Classification of domestic violence Persian textual content in social media based on topic modeling and ensemble learning
Source: Heliyon. 2024 Oct 29;10(22):e39953. doi: 10.1016/j.heliyon.2024.e39953 (PMC11583712; doi:10.1016/j.heliyon.2024.e39953)
Supplement: Multimedia component 2 [file mmc2.docx]

Appendix 2

| Text | **Translated to English** | **Topic Probabilities** | **Topic Label** |
| --- | --- | --- | --- |
| دخترهای هراتی را برای ازدواج نمی‌برند؛ برای تجاوز می‌برند؛ از واژه‌ها درست استفاده کنید | They don't take Herati girls for marriage; They take for rape; Use the right words | [(0, 0.15944645),  (1, 0.07796201),  (2, 0.41704434),  (3, 0.15596494),  (4, 0.18958229)] | 2 |
| به نظرم تقصیر اون دختر بچه 17 ماهه س که بهش تجاوز شده میخواست پوشش مناسب داشته باشه چون مرد اگه تحریک نشه که مریضه | In my opinion, it is the fault of the 17-month-old girl who was raped. She should have proper clothing because if the man is not provoked, he is sick. | [(0, 0.22296144),  (1, 0.056141656),  (2, 0.06593111),  (3, 0.056065265),  (4, 0.59890056)] | 4 |
| خیلی از افرادی که بهشون تجاوز شده و میان پزشکی قانونی نمیدونن چقدر اون نمونه‌ی اسپرم مهمه؛ نمونه برداری از روی سطح پوست و لباس، تا زمانی که شسته نشه، نمونه از مقعد ۳ روز و از واژن تا ۵ روز بعد از اون اتفاق امکان پذیره. | Many people who have been raped and among forensic doctors do not know how important that sperm sample is; It is possible to take samples from the surface of the skin and clothes, as long as it is not washed, from the anus for 3 days and from the vagina for 5 days after the incident. | [(0, 0.03604904),  (1, 0.03552699),  (2, 0.6444617),  (3, 0.122011594),  (4, 0.16195065)] | 2 |
